# Impacts of fire on non-native plant recruitment in black spruce forests of interior Alaska

**DOI:** 10.1371/journal.pone.0171599

**Published:** 2017-02-03

**Authors:** Xanthe J. Walker, Matthew D. Frey, Alexandra J. Conway, Mélanie Jean, Jill F. Johnstone

**Affiliations:** 1 Center for Ecosystem Science and Society, Northern Arizona University, Flagstaff, Arizona, United States of America; 2 Department of Biology, University of Saskatchewan, Saskatoon, Saskatchewan, Canada; Pacific Northwest National Laboratory, UNITED STATES

## Abstract

Climate change is expected to increase the extent and severity of wildfires throughout the boreal forest. Historically, black spruce (*Picea mariana* (Mill.) B.S.P.) forests in interior Alaska have been relatively free of non-native species, but the compounding effects of climate change and an altered fire regime could facilitate the expansion of non-native plants. We tested the effects of wildfire on non-native plant colonization by conducting a seeding experiment of non-native plants on different substrate types in a burned black spruce forest, and surveying for non-native plants in recently burned and mature black spruce forests. We found few non-native plants in burned or mature forests, despite their high roadside presence, although invasion of some burned sites by dandelion (*Taraxacum officinale*) indicated the potential for non-native plants to move into burned forest. Experimental germination rates were significantly higher on mineral soil compared to organic soil, indicating that severe fires that combust much of the organic layer could increase the potential for non-native plant colonization. We conclude that fire disturbances that remove the organic layer could facilitate the invasion of non-native plants providing there is a viable seed source and dispersal vector.

## Introduction

Recent changes in climate are expected to increase the extent and severity of wildfires throughout the boreal forest and may alter rates of colonization by non-native plants. Historically, boreal forests in interior Alaska have been relatively free of non-native species [1, 2], but the diversity and range of non-native plants has been increasing since the 1940s [3], and this may be accelerated by climate change and an altered fire regime. Climate warming may have direct effects on non-native plant recruitment by alleviating the constraints that cold temperatures and short growing seasons place on the growth and recruitment of non-native species originating in warmer climates [4]. Direct climate effects may interact with disturbance effects in determining invasion potential, as recent studies of non-native plants in boreal habitats suggest that disturbances by fire can facilitate invasion [2, 5]. Specifically, fire consumption of the soil organic layer in black spruce forests dramatically alters seedling recruitment success [6] and could facilitate the colonization and spread of non-native species. Fires that remove the canopy and cause warming of the soils due to increased solar radiation [7] may also facilitate colonization by non-native plants intolerant of colder soils. Furthermore, fires are known to increase soil nutrient availability [6] and increase rates of above ground productivity [7], which in turn support non-native plant establishment.

On a global scale, the spread of non-native plants can have negative economic [8] and environmental [9] impacts. For example, non-native plants can significantly impact ecosystem functioning, by displacing native species, altering soil nutrient cycles and hydrology, changing local fire regimes, and reducing native biodiversity [10]. Economically, the impacts and control of non-native plants have been estimated to cost approximately $35 billion annually in the United States [8]. Previous surveys conducted throughout interior Alaska have found high occurrences of non-native plants on roadsides [5, 11]. The roadside presence of non-native plants could enable dispersal into recently burned forest, which may negatively affect ecosystem structure and function if they are able to establish and persist in the boreal forest [4].

Here we used a combination of observational and experimental field studies to examine the effects of fire on current distributions and potential recruitment of non-native plants in Alaskan black spruce forests. As fire activity in boreal regions is expected to increase in response to climate change [12], it is important to understand the role that fire plays in non-native plant colonization to manage and direct efforts to control non-native plants in boreal forests.

## Materials and methods

### Study area

Our study area was located in interior Alaska, an area of boreal forest bounded by the Brooks Range to the north (~67°N) and the Alaska Range to the south (~63°N). Interior Alaska experiences a continental climate with large annual temperature fluctuations and is underlain by discontinuous permafrost [13]. Black spruce (*Picea mariana* (Mill.) B.S.P.) is the dominant forest type and is characterized by extensive moss cover, a sparse layer of tall shrubs, a well-developed layer of low shrubs, and a canopy of black spruce trees [1]. Fire is the primary disturbance agent in these forests, and increases in fire frequency, extent, severity, and fire season length have already occurred and are expected to continue in response to warming and drying climatic conditions [12, 14].

### Seeding experiment

We conducted seeding trials in 2012 in a recently burned (2004) black spruce forest in the Caribou-Poker Creeks Research Watershed (CPCRW), located approximately 50 kilometers north of Fairbanks, Alaska along the Steese Highway (65.14230 °N, 147.46828 °W). The Bonanza Creek LTER provided permission to conduct seeding trials at CPCRW. Three invasive plants commonly found in interior Alaska were chosen for the seeding experiment: *Vicia cracca* L. (bird vetch), *Melilotus officinalis* (sweet clover), and *Taraxacum officinale* G.H. Weber ex Wiggers (common dandelion). Each of the species chosen could potentially affect boreal ecosystem structure and function and are of concern based on the invasiveness ranking system for non-native plants in Alaska [15]. Both *M*. *officinalis* and *V*. *cracca* are nitrogen-fixing and therefore have a high potential for altering soil conditions and nutrient cycling [2, 16, 17] and/or facilitating the establishment of other invasive species [2, 18]. *T*. *officinale* is highly competitive in early-successional communities and can lead to a reduction of native species or individuals [15, 19]. Furthermore, these species range in seed size (estimated in the lab from the weight of 100 seeds), invasiveness, and life-history strategies, all of which might impact their ability to germinate and establish in a post-fire environment. Specifically, we expected larger sized seeds, with their greater carbohydrate reserves, to have a broader tolerance of seedbed conditions and higher germination rates in the field [20].

Seeds were collected in September 2011 from established populations in Fairbanks, Alaska, and stored in a freezer prior to seeding. We conducted laboratory trials with twenty seeds of each species to determine if scarification treatments would maximize germination. We applied mechanical scarification to *M*. *officinalis* by gently rubbing seeds between two pieces of sand paper [21] as this maximized germination (65% viability). Percussive scarification maximized germination of *V*. *cracca* (65% viability) and was applied by shaking seeds in an Erlenmeyer flask [21]. Germination of *T*. *officinale* (60% viability) seed was unaffected by scarification treatments and were therefore left untreated.

We established 10 experimental blocks, located at 10 m intervals along a 100 m transect. Each block consisted of three substrate treatments (plot size = 15 x 15 cm)), representing the common post-fire seedbeds of: regenerating moss (largely *Ceratodon purpureus* (Hedw.) Brid and *Polytrichum juniperinum* Hedw.), charred organic layer, and exposed mineral soil. As there was no exposed mineral soil in the burned forest at the time of the experiment, we removed surface organic material by hand to create mineral soil plots. We removed all shrubs and regenerating tree saplings within 50 cm of the plots to better emulate early post-fire conditions and control for potential effects of shading. We covered each plot with a mesh cage of galvanized metal (mesh size = 0.5 cm) to prevent small animals from removing seeds or seedlings. Ten seeds of *V*. *cracca* (0.012 g·seed^-1^), *M*. *officinalis* (0.0018 g·seed^-1^), and *T*. *officinale* (0.00081 g·seed^-1^) were sprinkled on the surface of each plot on 4 June 2012, to simulate natural dispersal. We counted total germinates on 30 July 2012, and removed all seedlings, visible seeds, and the surface substrate from the site to ensure that no non-native propagules remained.

### Non-native plant survey

In July 2012 we surveyed non-native plants in recently burned (6–8 years since fire) and mature (>60 years since fire) black spruce forests located along two major roadways in interior Alaska. Fifty sites (25 burned, 25 mature) were surveyed adjacent to the Dalton highway north of the Yukon River (burn year 2004), and 16 (8 burned, 8 mature) sites were surveyed adjacent to the Parks highway south of Nenana (burn year 2006) (see S1 and S2 Tables for site locations of burned and mature sites, respectively). We first identified all areas with non-native plant species along the road, thus restricting our surveys to areas that we could be confident had a propagule source in the road corridor. Transect locations for surveys of adjacent burned or mature forest were then randomly selected from these areas with a minimum distance of 100 m between locations along the road.

At the roadside of each transect, a 1 m^2^ plot adjacent to the road was used to visually estimate non-native plant abundances. Non-native plants were recorded as having zero (0 individuals/m^2^), low (<25 individuals/m^2^), medium (25–50 individuals/m^2^), or high (>50 individuals/m^2^) presence. These estimates of abundance were used as an indicator of propagule pressure. We then ran a 100 x 2 m belt transect perpendicular from the forest edge into the forest. The forest edge was located by visually assessing a shift from the disturbed roadside to typical forest understory plant communities. Abundance of all non-native plants within the belt transect was recorded and distance into the forest stand was recorded for sites along the Dalton Highway. Although not reported here, environmental, understory, and seedling data were also collected at each transect [22].

### Statistical analysis

All data analyses were performed using the R statistical package version 3.0.2 [23]. R code and the associated raw datasets are available in S1 File and S3–S5 Tables, and are part of the data archived for this project in the LTER database (http://www.lter.uaf.edu/data/data-detail/id/587 and http://www.lter.uaf.edu/data/data-detail/id/588).To assess the effect of substrate type on seed germination counts from the seeding experiment we used a Kruskal-Wallis rank sum test. This test ranks the data and is therefore more robust to small samples and the non-normally distributed nature of our data than the parametric alternative [24]. If differences were detected between substrate types, we used a pairwise Wilcoxon rank sum test with a Bonferroni adjustment to compare seed germination among substrate types within the burned forest for each of the three seeded species.

Based on non-native plant surveys we calculated the frequency of non-native plant occurrences for road sides and transects in burned and mature forest transects. We used presence and absence of non-native plants and determined if the probability of encountering a non-native species is the same whether in an unburned forest transect, a burned forest transect, or on the roadside surveys adjacent to the unburned and burned stands using a Fisher’s exact test. We then used a pairwise post-hoc Fisher’s test in the package “fifer” [25] to complete pairwise comparisons between surveyed areas. To test if the dispersal distribution of non-native plants in the burned stands was evenly distributed along transects with increasing roadside distance, we calculated the frequency of non-native plant presence within four distance categories (0–25, 25–50, 50–75, and 75–100 meters from the road edge). We then completed a chi-square test of these frequencies compared to an even frequency distribution. This analysis largely tested the distribution of *T*. *officinale*, given the low abundance of other species. When non-native plants were present, we assumed that dispersal was from the roadside and calculated the mean density and distance into the forest that the plant had dispersed.

## Results

### Seeding experiment

**Fig 1:**
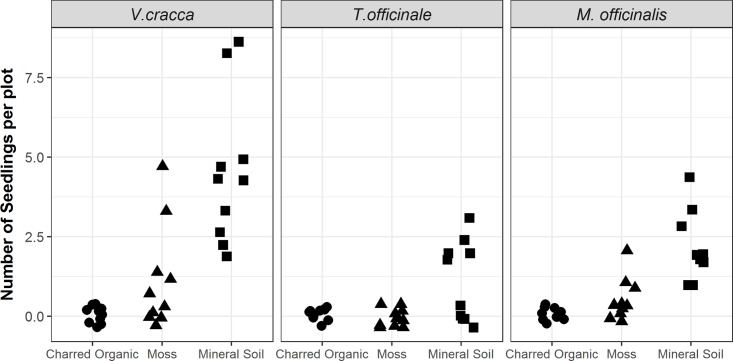
Seedling counts of *V*.*cracca*, *T*.*officinale*, and *M*.*officinalis* from germination trials on charred organic, moss, and mineral soil substrates within a burned forest (n = 10 plots). Each plot was seeded with 10 seeds of the assigned species. Note points are slightly offset for visualization.

Germination of all three seeded species were affected by substrate type (*p*<0.001). Germination was consistently highest on mineral soil (Fig 1 and Table 1), and no species had successful germination on residual, charred organic soil (Fig 1). *V*. *cracca*, which had the largest seeds of the three species, had the highest germination rates on both moss and exposed mineral soil (Fig 1 and Table 1).

**Table 1:** Results of Kruskal-Wallis rank sum test and pairwise Wilcoxon rank sum test between substrate types for germination trials of *V*.*cracca*, *T*.*officinale*, and *M*.*officinalis* on charred organic, moss, and mineral soil substrates within a burned forest (n = 10). Bold text indicates significant results.

	*V*.*cracca*	*T*.*officinale*	*M*.*officinalis*
Kruskal-Wallis rank sum test	*X*^*2*^ = 20.63, *p*<0.001	*X*^*2*^ = 11.53, *p*<0.005	*X*^*2*^ = 21.39, *p*<0.001
Charred Organic vs. Moss	***p* = 0.044**	NA*	*p* = 0.232
Charred Organic vs. Mineral Soil	***p*<0.001**	***p* = 0.029**	***p*<0.001**
Moss vs. Mineral Soil	***p* = 0.007**	***p* = 0.029**	***p*<0.005**

*Unable to calculate exact p-value for ties.

### Non-native plant survey

We found roadside occurrences of eight non-native species (*M*. *officinalis*, *T*. *officinale*, and *V*. *cracca*, as well as *Crepis tectorum*, *Hordeum jubatum*, *Plantago major*, *Matricaria discoidea*, and *Chenopodium album* along the Dalton and Parks Highway adjacent to both burned and mature forest stands (Table 2). *M*. *officinalis*, and *T*. *officinale* were encountered often along the roadside, while all other species were infrequently encountered in the roadside, and were never observed colonizing burned stands in this study. The probability of encountering an invasive plant was significantly different among burned forest, unburned forest, and roadsides adjacent to burned and unburned forests (*p*<0.001). Non-native plants were not observed in any of the 33 mature forest stands and observed in 11 of the 33 surveyed burned stands (eight in the Dalton highway study region, three in the Parks highway study region). *M*. *officinalis* was present along the roadside for all sites surveyed, however it was only observed in one of the burned stands along the Dalton highway. *C*. *tectorum* was observed in one burned site along the Dalton highway despite being absent from the roadside adjacent to this site, and was seldom encountered on the roadside. Of all the non-native species, *T*. *officinale* had the highest occurrence in burned stands and was present in eight sites (33%) on the Dalton Highway and three (37.5%) along the Parks highway. Along the Dalton highway, non-native plants, specifically *T*. *officinale*, was evenly distributed with distance along the 100 m transects (*X*^*2*^ = 3.23, df = 3, p = 0.361) with 26%,19%, 20% and 35% of its occurrences found at 0–25, 25–50, 50–75, and 75–100 m from the roadside, respectively. When present *T*. *officinale* had a mean distance of 47.26 m (±2.50 SE) into burned stands and density of 4.26 (±0.52 SE) individuals/m^2^.Other species that are considered to be invasive plants of concern in Alaska [15], such as *V*. *cracca*, *H*. *jubatum*, *M*. *discoidea*, *C*. *album*, and *P*. *major* were infrequently encountered in the roadside, and were never observed colonizing burned stands in this study.

**Table 2:** Proportion of road survey transects with absent, low, medium, and high roadside densities of non-native plants adjacent to burned and unburned transects along the Dalton and Parks Highways.

Forest type	Highway	Density	*Taraxacum officinale*	*Melilotus officinalis*	*Crepis tectorum*	*Hordeum jubatum*	*Mastricia discoidea*	*Chenopodium album*	*Plantago major*	*Vicia cracca*
Burned	Dalton (n = 25)	Absent	0.080	0	0.840	0.960	0.960	0.960	0.960	0.960
		Low	0.640	0.280	0.160	0.040	0.040	0.040	0.040	0.040
		Medium	0.240	0.400	0	0	0	0	0	0
		High	0.040	0.320	0	0	0	0	0	0
	Parks (n = 8)	Absent	0.500	0	1	1	1	1	0.875	1
		Low	0.500	0.875	0	0	0	0	0.125	0
		Medium	0	0.125	0	0	0	0	0	0
		High	0	0	0	0	0	0	0	0
Unburned	Dalton (n = 25)	Absent	0	0	0.840	0.760	1	1	0.920	0.800
		Low	0.60	0.240	0.160	0.160	0	0	0.080	0.120
		Medium	0.280	0.400	0	0.080	0	0	0	0
		High	0.120	0.360	0	0	0	0	0	0.080
	Parks (n = 8)	Absent	0.500	0	1	1	1	1	0.875	1
		Low	0.500	0.875	0	0	0	0	0.125	0
		Medium	0	0.125	0	0	0	0	0	0
		High	0	0	0	0	0	0	0	0

*Note*: Density categories are: low <25 individuals/m^2^; medium 25–50 individuals/m^2^, high >50 individuals/m^2^

## Discussion

This study provides evidence that fire disturbance has not facilitated aggressive non-native plant colonization in black spruce forest in interior Alaska. We found no non-native plants in mature black spruce forests and only a third of the recently burned forests examined were colonized by *T*. *officinale* despite high roadside presence of non-native plants along both Dalton and Parks Highways. However, in our seeding experiment, surface organic layers and their effect on seedbed conditions were key factors influencing seeding success. Thus, it is possible that fire disturbances that remove the organic layer could facilitate the invasion of non-native plants providing there is a viable seed source and dispersal vector.

In our seeding experiment, germination rates were consistently higher on exposed mineral soil than on charred organic layer or moss. These results are consistent with other studies in the boreal forest, where disturbances that remove the surface organic layer and expose mineral soil favor the colonization of non-native plants [26] and recruitment of native tree seedlings [6]. Independent of substrate type, *V*. *cracca* had higher germination rates (on a per seed basis) than either *T*. *officinale* or *M*. *officinalis*, which is likely due to variations in seed characteristics. *T*. *officinale* and *M*. *officinalis* produce a large volume of small seeds [27, 28], whereas *V*. *cracca* produces fewer, larger seeds [29]. The small seeded *T*. *officinale* and *M*. *officinalis* may not have had sufficient carbohydrate reserves to establish from seed when moss and litter provide shade and prevent rapid root penetration into mineral soil, compared to the large seeded *V*. *cracca*. This supports the concept that large-seeded plants have a broader tolerance of seedbed conditions that permit seedling establishment [1, 20].

In our non-native plant surveys, *T*. *officinale* was the dominant invading non-native species, despite the higher roadside occurrence of *M*. *officinalis* and higher experimental germination rates of *V*. *cracca*. The presence of *T*. *officinale* in burned stands may be attributed to small, wind dispersed seeds that can disperse several hundred meters from the source [19, 30, 31], increasing potential colonization of preferred substrate types with less residual organic layer cover [22]. Indeed, *T*. *officinale* requires open disturbed soil to establish [30], such as those created following severe fires [30]. In contrast, *V*. *cracca* disperses by explosive pods or rhizomes and relies on animal vectors for long distance dispersal [15, 29, 30, 32].

Large seeds and low dispersal capacity may explain the rare presence of *V*. *cracca* in our roadside surveys and its absence from burned stands. In contrast, *M*. *officinalis* was abundant along roadsides and is known to disperse well by water and human vectors in disturbed habitats associated with roads and rivers [2].Although the roadside presence of *M*. *officinalis* was high in our surveys, the occurrence of *M*. *officinalis* into burned areas was low (<4%) on the Dalton Highway and absent along the Parks Highway. *M officinalis* is easily dispersed by water, and grows best on well drained exposed mineral soils that are alkaline to slightly acidic [2, 15, 16, 19]. These characteristics correspond to those of roadsides and roadside ditches, which are mostly made from sand and gravel from floodplains or rock quarries [2]. Upland black spruce soils in interior Alaska are acidic (pH 3.8–4.5) [1] even following a severe fire [33], which is much lower than the optimum pH for *M*. *officinalis* growth [2]. Dispersal limitations, in association with a lack of suitable seedbed for colonization, may explain the absence of *M*. *officinalis* in burned stands despite the high roadside propagule pressure. These results highlight the role of dispersal in shaping patterns of non-native species establishment, as we observed the wind-dispersed *T*. *officinale* colonizing more new territory while other species with higher potential germination, such as *M*. *officinalis* and *V*. *cracca*, remained largely absent from burned and unburned stands.

This study supports the general observation that non-native plant establishment remains low in natural boreal forests [12, 29]. Even in disturbed forests, we found limited spread of non-native plant species into recently burned areas along both the Dalton and Parks Highways, despite abundant roadside populations of *T*. *officinale* and *M*. *officinalis*. Moreover, the diversity of non-native plants found along the roadways was low, and other species such as *V*. *cracca*, *H*. *jubatum*, *M*. *discoidea*, *C*. *album*, and *P*. *major* were rarely encountered in our surveys. Current low densities of non-native species in Alaskan boreal forests are likely maintained by a combination of low propagule pressure away from human population centers and poor seedbeds that restrict colonization of burned and mature forests. Our seeding experiments indicate that fire disturbances that remove the organic layer could facilitate the invasion of non-native plants providing there is a dispersal vector for viable seeds. Strategic monitoring of non-native plant spread could benefit from mapping of fire severity and other disturbances that may remove the soil organic layer in black spruce forests, especially in those areas where propagule pressure is high.

## Supporting information

S1 File R code for data analysis of seeding experiment and non-native plant survey data. (DOCX)

S1 Table Survey site coordinates for burned stands adjacent to the Dalton (DC) and Parks (NE) highways. (DOCX)

S2 Table Survey site coordinates for mature stands (>60years since fire) adjacent to the Dalton (DC) and Parks (NE) highways. (DOCX)

S3 Table Presence of *Taraxacum officinale* (TAOF), *Crepis tectorum* (CRTE), and *Melilotus officinalis* (MEOF) associated with distance (0–100 meters) from the highway for each site. (TXT)

S4 Table Roadside presence (high, medium, or low) or absence of *Taraxacum officinale* (TAOF), *Melilotus officinalis* (MEOF), *Crepis tectorum* (CRTE), and *Hordeum jubatum* (HOJU), *Mastricia discoidea* (MADI), *Chenopodium album* (CHAL), *Plantago major* (PLMA), and *Vicia cracca* (VICR) for each site along each highway (DC = Dalton, NE = Parks) and stand type (BU = burned, UB = unburned). (TXT)

S5 Table Experiment seeding counts of *Vicia cracca* (VICR), *Taraxacum officinale* (TAOF), and *Melilotus officinalis* (MEOF) for each of the applied treatment categories. (TXT)
