# Supplementary material for: Impacts of fire on non-native plant recruitment in black spruce forests of interior Alaska
Source: PLoS One. 2017 Feb 3;12(2):e0171599. doi: 10.1371/journal.pone.0171599 (PMC5291358; doi:10.1371/journal.pone.0171599)
Supplement: S1 File — (DOCX) [file pone.0171599.s001.docx]

S3 File. R code for data analysis of seeding experiment and non-native plant survey data.

#########################3

###################################seeding experiment code

data<-read.table("S5 Table.txt",header=T, na.strings=c("", "NA"), sep="\t")

kruskal.test(data2$VICR~data2$Treatment)

pairwise.wilcox.test(data2$Vic, data2$Treatment, p.adjust.method = "bonferroni")

kruskal.test(data2$TAOF~data2$Treatment)

pairwise.wilcox.test(data2$Tar, data2$Treatment, p.adjust.method = "bonferroni")

kruskal.test(data2$MEOF~data2$Treatment)

pairwise.wilcox.test(data2$Mel, data2$Treatment, p.adjust.method = "bonferroni")

####################non-native plant surveys

########################################3

################## fisher's test for presence and absence - counts of invasive species in different areas

present<-c(11,0,33,33)

absent<-c(22,33,0,0)

matrix<-rbind(present,absent)

matrix

colnames(matrix)<-c("burned.forest","unburned.forest","burned.road", "unburned.road")

fisher.test(matrix)

###install and use “fifer” package version 1.0 for post.hoc test

library(fifer)

chisq.post.hoc(t(matrix), test="fisher.test")

###############proportions calculated for each distance category

data<-read.table("S3 Table.txt",header=T, na.strings=c("", "NA"), sep="\t")

attach(data)

names(data)

###install dplyr package version 0.5.0 for data manipulation

library(dplyr)

data1<-mutate(data, invasives=TAOF+CRETE+MEOF)

names(data1)

data1$distcat<-cut(data1$DIST, seq(0,100,25), labels=c(1:4))

head(data1)

data1<-aggregate(data1[,c(6)], by=list(data1$distcat), FUN=sum)

data1

############change into proportions by dividing count by total and multiply by 100

########### for example (148/566*100 = 26.1)

count<-matrix(c(26.1,18.9,19.8,35.2, 25, 25, 25, 25), ncol=2)

chisq.test(count)
