# Supplementary material for: Impacts of fire on non-native plant recruitment in black spruce forests of interior Alaska
Source: PLoS One. 2017 Feb 3;12(2):e0171599. doi: 10.1371/journal.pone.0171599 (PMC5291358; doi:10.1371/journal.pone.0171599)
Supplement: S1 Table — (DOCX) [file pone.0171599.s002.docx]

| Site | Latitude | Longitude | Elevation (masl) |
| --- | --- | --- | --- |
| DCBU1 | 66.07639 | 150.16679 | 222 |
| DCBU2 | 66.20036 | 150.22331 | 366 |
| DCBU3 | 66.20187 | 150.22523 | 362 |
| DCBU4 | 66.20366 | 150.22728 | 367 |
| DCBU5 | 66.15855 | 150.19194 | 272 |
| DCBU6 | 66.16156 | 150.19608 | 271 |
| DCBU7 | 66.16391 | 150.19922 | 282 |
| DCBU8 | 66.13938 | 150.17259 | 249 |
| DCBU9 | 65.97484 | 149.99731 | 190 |
| DCBU10 | 66.09384 | 150.15469 | 233 |
| DCBU11 | 66.14643 | 150.17847 | 245 |
| DCBU12 | 66.14991 | 150.18213 | 268 |
| DCBU13 | 66.16811 | 150.20499 | 296 |
| DCBU14 | 66.16586 | 150.20183 | 289 |
| DCBU15 | 66.07481 | 150.16632 | 222 |
| DCBU16 | 66.07317 | 150.16588 | 216 |
| DCBU17 | 66.10873 | 150.15900 | 222 |
| DCBU18 | 66.07986 | 150.16766 | 224 |
| DCBU19 | N/A | N/A | N/A |
| DCBU20 | 65.89957 | 149.76402 | 107 |
| DCBU21 | 65.92149 | 149.82535 | 147 |
| DCBU22 | 65.90988 | 149.78592 | 127 |
| DCBU23 | 65.91006 | 149.78296 | 125 |
| DCBU24 | 65.90690 | 149.77855 | 110 |
| DCBU25 | 65.90804 | 149.78073 | 112 |
| NEBU1 | 64.38049 | 149.02008 | 142 |
| NEBU2 | 64.39147 | 149.01340 | 144 |
| NEBU3 | 64.39700 | 149.01266 | 145 |
| NEBU4 | 64.39924 | 149.01303 | 139 |
| NEBU5 | 64.40218 | 149.01520 | 137 |
| NEBU6 | 64.47121 | 149.06064 | 128 |
| NEBU7 | 64.48678 | 149.07846 | 127 |
| NEBU8 | 64.49713 | 149.06995 | 123 |

S1 Table. Survey site coordinates for burned stands adjacent to the Dalton (DC) and Parks (NE) highways.

Survey coordinates recorded under WGS84 Map Datum.
