# Supplementary material for: Impacts of fire on non-native plant recruitment in black spruce forests of interior Alaska
Source: PLoS One. 2017 Feb 3;12(2):e0171599. doi: 10.1371/journal.pone.0171599 (PMC5291358; doi:10.1371/journal.pone.0171599)
Supplement: S2 Table — (DOCX) [file pone.0171599.s003.docx]

S2 Table. Survey site coordinates for mature stands (>60years since fire) adjacent to the Dalton (DC) and Parks (NE) highways.

| Site | Latitude | Longitude | Elevation (masl) |
| --- | --- | --- | --- |
| DCBU1 | 66.17370 | 150.21123 | 259 |
| DCBU2 | 65.95683 | 149.94066 | 178 |
| DCBU3 | 66.02691 | 150.12350 | 178 |
| DCBU4 | 66.06051 | 150.15959 | 249 |
| DCBU5 | 66.06179 | 150.16135 | 238 |
| DCBU6 | 66.18194 | 150.21223 | 308 |
| DCBU7 | 66.18375 | 150.21219 | 295 |
| DCBU8 | 66.09705 | 150.15146 | 230 |
| DCBU9 | 66.09552 | 150.15303 | 233 |
| DCBU10 | 66.09384 | 150.15469 | 233 |
| DCBU11 | 66.06389 | 150.16145 | 249 |
| DCBU12 | 66.06561 | 150.16216 | 228 |
| DCBU13 | 66.05901 | 150.15883 | 257 |
| DCBU14 | 66.07899 | 150.16692 | 215 |
| DCBU15 | 66.08084 | 150.16667 | 219 |
| DCBU16 | 66.09255 | 150.15643 | 239 |
| DCBU17 | 66.13342 | 150.16785 | 218 |
| DCBU18 | 66.06775 | 150.15817 | 260 |
| DCBU19 | 66.05620 | 150.15791 | 256 |
| DCBU20 | 65.93495 | 149.85870 | 180 |
| DCBU21 | 65.93378 | 149.85597 | 147 |
| DCBU22 | 65.93592 | 149.86128 | 186 |
| DCBU23 | 65.93642 | 149.86366 | 176 |
| DCBU24 | 65.93868 | 149.87344 | 171 |
| DCBU25 | 65.93929 | 149.87682 | 161 |
| NEBU1 | 64.08484 | 149.21709 | 321 |
| NEBU2 | 64.09386 | 149.22726 | 310 |
| NEBU3 | 64.10535 | 149.23541 | 311 |
| NEBU4 | 64.26083 | 149.16292 | 197 |
| NEBU5 | 64.26373 | 149.15083 | 170 |
| NEBU6 | 64.28052 | 149.10594 | 177 |
| NEBU7 | 64.29224 | 149.08817 | 176 |
| NEBU8 | 64.30039 | 149.07938 | 151 |

Survey coordinates recorded under WGS84 Map Datum.
